# Unmet needs in Cushing’s syndrome: the patients’ perspective

**DOI:** 10.1530/EC-22-0027

**Published:** 2022-05-27

**Authors:** Elena Valassi, Iacopo Chiodini, Richard A Feelders, Cornelie D Andela, Margueritta Abou-Hanna, Sarah Idres, Antoine Tabarin

**Affiliations:** 1 Endocrinology Department, Germans Trias i Pujol Hospital and Research Institute, Badalona, Barcelona, Spain; 2 Centro de Investigación Biomédica en Red de Enfermedades Raras (CIBERER), Madrid, Spain; 3 Universitat Internacional de Catalunya (UIC), Barcelona, Spain; 4 IRCCS, Istituto Auxologico Italiano, Milan, Italy; 5 Department of Medical Biotechnology and Translational Medicine, University of Milan, Milan, Italy; 6 Division of Endocrinology, Erasmus Medical Centre, Rotterdam, Netherlands; 7 Division of Endocrinology and Centre for Endocrine Tumours, Leiden University Medical Centre, Leiden, Netherlands; 8 HRA Pharma Rare Diseases, Châtillon, France; 9 Department of Endocrinology and INSERM U862 University and CHU of Bordeaux, Pessac, France

**Keywords:** Cushing’s syndrome, management, patient experience, patient survey, quality of life

## Abstract

**Background:**

Cushing’s syndrome (CS) is a rare condition of chronically elevated cortisol levels resulting in diverse comorbidities, many of which endure beyond successful treatment affecting the quality of life. Few data are available concerning patients’ experiences of diagnosis, care and persistent comorbidities.

**Objective:**

To assess CS patients’ perspectives on the diagnostic and care journey to identify unmet therapeutic needs.

**Methods:**

A 12-item questionnaire was circulated in 2019 by the World Association for Pituitary Organisations. A parallel, 13-item questionnaire assessing physician perceptions on CS patient experiences was performed.

**Results:**

Three hundred twenty CS patients from 30 countries completed the questionnaire; 54% were aged 35–54 and 88% were female; 41% were in disease remission. The most burdensome symptom was obesity/weight gain (75%). For 49% of patients, time to diagnosis was over 2 years. Following treatment, 88.4% of patients reported ongoing symptoms including, fatigue (66.3%), muscle weakness (48.8%) and obesity/weight gain (41.9%). Comparisons with delay in diagnosis were significant for weight gain (*P* = 0.008) and decreased libido (*P* = 0.03). Forty physicians completed the parallel questionnaire which showed that generally, physicians poorly estimated the prevalence of comorbidities, particularly initial and persistent cognitive impairment. Only a minority of persistent comorbidities (occurrence in 1.3–66.3%; specialist treatment in 1.3–29.4%) were managed by specialists other than endocrinologists. 63% of patients were satisfied with treatment.

**Conclusion:**

This study confirms the delay in diagnosing CS. The high prevalence of persistent comorbidities following remission and differences in perceptions of health between patients and physicians highlight a probable deficiency in effective multidisciplinary management for CS comorbidities.

## Introduction

Cushing’s syndrome (CS) is a morbid endocrine condition due to prolonged exposure to high circulating cortisol levels (1, 2, 3). Hypercortisolism may cause irreversible physical and psychological changes in several tissues, leading to debilitating morbidities which persist over the long term after the resolution of excessive hormone levels, such as cardiovascular complications, metabolic and skeletal disorders, infections and neuropsychiatric disturbances (3, 4). Even patients who have been biochemically ‘cured’ for over 10 years have a residual overall higher risk of mortality, mostly from circulatory disease and diabetes (5). Moreover, people with a history of CS suffer from impaired quality of life (QoL) (6). Several studies suggest that the prevalence of persistent comorbidities is correlated with the duration of exposure to cortisol excess (7, 8). However, as the signs and symptoms of CS overlap with common diseases such as the metabolic syndrome and depression, the time taken to diagnose CS is often long, resulting in a significant number of patients with persistent sequelae and impairments in QoL (6, 9).

Given the burden of the disease, ideal CS treatment would include early diagnosis, curative surgery and multidisciplinary care of comorbidities both pre- and post-cure of CS, including the psychological dimension of the patient’s disease experience (10). Few data are available about patients’ perceptions of the medical journey from first symptoms to diagnosis, treatment and follow-up. The aim of this study was, therefore, to explore CS patients’ experiences of symptoms, diagnosis, care and treatment satisfaction around the world and to compare patients’ perceptions of CS with those of physicians.

## Methods

### Patient questionnaire design

A 12-item patient questionnaire was developed based on the generally understood clinical characteristics and symptomology of CS, aiming to assess patients’ experiences of symptoms, diagnosis, care and treatment satisfaction (1, 2) (Supplementary File 1, see section on supplementary materials given at the end of this article). The questionnaire was initially offered in English and made available via the SurveyMonkey online platform from March to May 2019. The survey was completed anonymously and required no specific participant identification or any details that could be used to identify individual participants. In addition to basic demographics (i.e. country of residence, sex, age and highest educational level attained), the questionnaire asked ten multiple-choice and two open questions. The survey was shared by the World Association for Pituitary Organisations (WAPO), Adrenal Net, Cushing’s Support & Research Foundation and the Pituitary Foundation, as well as being distributed to local patient associations. As a second step, the questionnaire was translated into eight additional languages (French, Dutch, Spanish, Chinese, Portuguese, Italian and German) and was recirculated by the WAPO, Adrenal Net and China Hypercortisolism Patient Alliance to the different local patient associations for distribution in November 2019. As this was a non-interventional, anonymous patient survey, distributed by the patient associations themselves, and not initiated or funded by a research or educational institution, no ethical review was required. Written consent was obtained from each respondent after full explanation of the purpose and nature of the survey.

### Comparative physician survey

In addition, a 13-item physician questionnaire was developed to assess physicians’ perspectives on CS symptoms and comorbidities. This physician questionnaire was conducted by HRA Pharma Rare Diseases at the 2019 European Congress of Endocrinology, in Lyon, France. This anonymous questionnaire was completed by 40 qualified physicians. The responses from the patient survey were compared for context with the physicians’ estimates of the prevalence of CS symptoms and comorbidities. Although the physician questionnaire was conducted independently of the patient questionnaire, and used a different question structure, the comparison with the current patient questionnaire is included to further enrich and contextualise the patient responses.

### Data analysis

All responses and answers were collected, coded and analysed using Microsoft Excel. Data preparation involved removing duplicate answers, or where possible analysing and reclassifying qualitative responses reported as ‘other’, based on the accompanying details to new or existing response options.

### Statistical methodology

Complementary statistical analyses using SAS software were performed using the chi-square and Fisher tests, depending on the cell counts, to compare (i) the time between first symptoms and diagnosis and the persistence of symptoms and (ii) persistence of symptoms, with the specialities of the physicians currently treating the respondents. Frequency distribution of a particular variable was displayed and compared with the frequency distribution of the comparator variable. A significance level of 0.05 was applied.

## Results

### Demographic characteristics

Three hundred twenty patients from 30 countries completed the patient questionnaire, with 27% (*n*  = 87) coming from the United Kingdom and 14% (*n*  = 44) from the United States of America. More than half (53.7%, *n* = 172) of the patients were aged between 35 and 54 years, and 88.4% (*n*  = 283) were female. The majority of patients (53.1%, *n* = 170) had undergraduate or postgraduate qualifications (Table 1).

**Table 1:** Patient demographics.

Sex	*N* = 319^a^
Female	283 (88.4%)
Male	36 (11.3%)
Age group	*N* = 320
18–24 years	16
25–34 years	49
35–44 years	71
45–54 years	101
55–64 years	54
65–74 years	24
≥75 years	5
Region^b^	*N* = 320
Western Europe	222
North America	60
China	16
Australasia	14
South America	5
Africa	3
Education	*N* = 320
High school graduate/secondary education diploma	35%
Undergraduate degree	25.6%
Post-graduate degree	27.5%
Prefer not to say	10.6%
Time from first symptoms to diagnosis	*N* = 320
0–6 months	18.4%
6–12 months	15.6%
1–2 years	14.4%
2–3 years	18.4%
3–5 years	11.6%
5–10 years	8.4%
10–15 years	7.5%
15–20 years	0.9%
20+ years	1.9%
Unknown	2.8%

^a^One patient responded ‘non-binary’. ^b^Western Europe: United Kingdom (*n*  = 87), the Netherlands (*n*  = 38), France (*n*  = 37), Spain (*n*  = 12), Denmark (*n*  = 10), Norway (*n*  = 9), Germany (*n*  = 6), Italy (*n*  = 5), Ireland (*n*  = 4), Belgium (*n*  = 4), Poland (*n*  = 4), Sweden (*n*  = 2), Malta (*n*  = 2), Switzerland (*n*  = 1), Czech Republic (*n*  = 1); Africa: South Africa (*n*  = 1), Gabon (*n*  = 1), Zimbabwe (*n*  = 1); Australasia: Australia (*n*  = 8), New Zealand (*n*  = 6); South America: Colombia (*n*  = 2), Bolivia (*n*  = 1), Argentina (*n*  = 1), Brazil (*n*  = 1); North America: United States of America (*n*  = 44), Canada (*n*  = 13), Costa Rica (*n*  = 1), Mexico (*n*  = 1), Dominican Republic (*n*  = 1).

### Time to diagnosis

The time to diagnosis from first reporting of CS symptoms was declared to be within 2 years for 48.4% (*n*  = 155) (Table 1) and was over 2 years in 48.7% (*n*  = 156) and over 3 years in 30.3% (*n*  = 97).

### Initial symptoms

A broad range of signs and symptoms were initially noticed by patients, with weight gain, hirsutism or acne, fatigue, sleep disturbances, depressive symptoms, muscle weakness, anxiety and hypertension all being reported in over 50% of patients (Table 2). Obesity/weight gain was most commonly cited (75%, *n* = 240) as being burdensome. Fatigue, feelings of depression or mood problems, sleep disturbances, muscle weakness and hirsutism were also very commonly (>40%) mentioned as being burdensome. Burdensome symptoms classified as ‘other’ were rare (<1%) and included issues such as hormonal problems and dental problems.

**Table 2:** Patient-reported symptoms (multiple answers were possible).

	Symptoms first noticed (%)	Most burdensome perceived symptoms before diagnosis (%)
Weight gain	85.0	75.0
Hirsutism/acne	76.3	42.8
Fatigue	66.3	54.1
Sleep disturbances	64.4	41.9
Skin problems	64.7	21.3
Depression/mood problems	58.8	48.1
Muscle weakness	57.8	43.4
Anxiety	54.1	39.1
Hypertension	52.5	22.2
Loss of concentration	45.0	28.4
Memory problems	41.9	30.3
Menstrual disturbances	35.6	12.5
Decreased libido	32.5	12.5
Bone problems	23.1	14.4
Infections	23.8	10.3
Glucose intolerance	17.2	8.4
Blood clot	5.3	
Pain(s)	3.1	
Vision problems	2.8	
Headache	2.5	
Cravings	1.6	
Other	8.4	1.9

### Person who made the initial CS diagnosis

In 53.8% (*n*  = 172) of cases, an endocrinologist made the initial diagnosis of CS or prescribed the first screening tests, Table 3. General practitioners made 18.1% of diagnoses (*n*  = 58), in the remaining cases a diversity of other physicians directly or indirectly contributed to make the diagnosis, as indicated in Table 3. A small but noticeable number (5.6%, *n* = 18) of patients self-diagnosed and then convinced their physician to order the diagnostic tests.

**Table 3:** Patient perception of physician specialty.

Specialty	Person who made the initial diagnosis or suspected Cushing’s syndrome (%) (*n* = 320)	Physicians involved in the management of Cushing’s syndrome (%) (*n* = 320)
Endocrinologist	53.8	97.8
General practitioner/family doctor	18.1	56.3
Self-diagnosed	5.6	–
Hospital/emergency doctor	3.8	–
Internist	2.5	0.9
Gynecologist	1.9	14.1
Cardiologist	1.9	13.4
Bone specialist	1.9	14.1
Dermatologist	1.6	11.6
Haematologist	0.9	3.8
Ophthalmologist	0.9	3.1
Nurse	0.9	2.5
Radiologist	0.9	0.6
Family or friend	0.9	–
Psychiatrist or psycologist	0.9	23.4
Healer	0.6	2.2
Surgeon	0.6	–
Oncologist	0.3	6.6
Gastroenterologist	0.3	1.3
Neurologist	0.3	4.1
Others	1.6	–
Physiotherapist	–	14.4
Dietician	–	9.7
Neurosurgeon	–	8.1
Social worker	–	4.1
Ear, nose and throat specialist	–	1.6
Sports physician	–	1.3
Sleep specialist	–	0.9
Urologist	–	0.6
Orthopaedic surgeon	–	0.3

### Response to treatment

**Figure 1:**
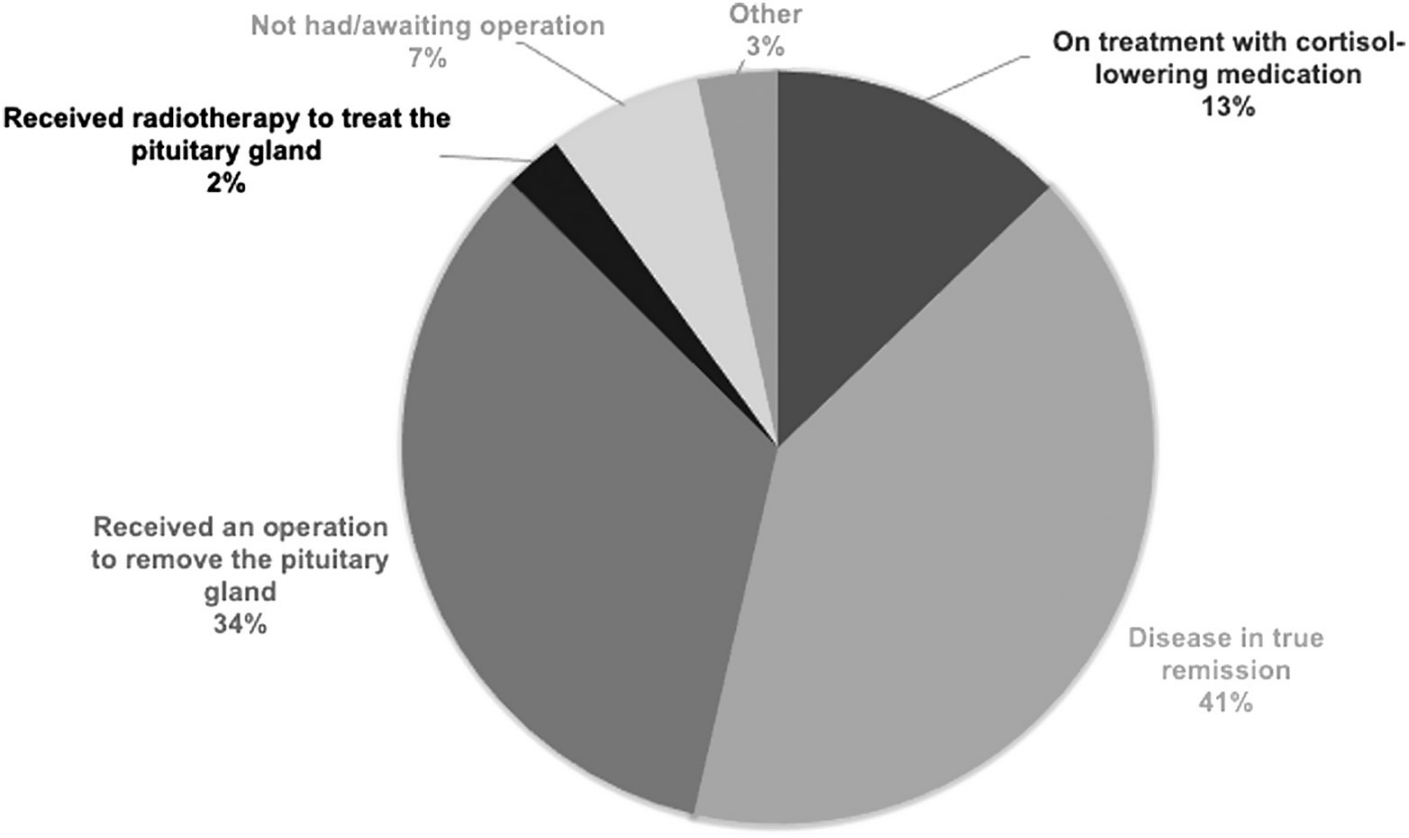
Patient description of their current clinical situation (*n* = 319). The category ‘Disease in true remission’ combines scores for ‘In remission (no treatment)’ (16.3%), ‘Received an operation to remove adrenal glands’ (22.9%) and ‘Treated with hydrocortisone’ (1.6%). One person did not complete the question.

At the time of answering the questionnaire, 55.8% (*n*  = 178) of patients were not in remission. 40.8% of patients (*n*  = 130) were in true biochemical remission (Fig. 1). This latter group was a composite including patients who responded: ‘In remission (no treatment)’ (16.3%, *n* = 52), ‘Received an operation to remove adrenal glands’ (22.9%, *n* = 73) and ‘Treated with hydrocortisone’ (1.6%, *n* = 5). Thirteen percent of the patients (*n*  = 41) were on cortisol-lowering treatment and 6.6% of the patients (*n*  = 21) had not had or were awaiting surgery. Following treatment for CS, 11.6% of the patients (*n*  = 37) reported having no further symptoms related to the condition, with 88.4% (*n*  = 283) still symptomatic. Of the total population (*n*  = 320), the most bothersome symptoms were fatigue (66.3%, *n* = 212), muscle weakness (48.8%, *n* = 156) and obesity/weight gain (41.9%, *n* = 134) (Table 4).

**Table 4:** Persistent symptoms.

Symptom	Persistent bothersome symptoms^a^ (%) (*n* = 320)	Treatment received for symptoms (%) (*n* = 320)
Fatigue	66.3	15.9
Muscle weakness	48.8	17.2
Weight gain	41.9	8.4
Depression, mood problems	36.9	28.8
Poor concentration	35.9	4.1
Memory problems	33.8	5.6
Sleep problems	33.1	14.1
Anxiety	30.6	14.7
Decreased libido	25.3	4.1
Bone problems	19.1	21.9
Hypertension	18.4	29.4
Hirsutism	17.5	4.1
Skin problems	16.6	6.9
Glucose intolerance	8.8	10
Menstrual problems	9.1	4.7
Infections	7.2	4.7
Blood clot	3.8	2.2
Acne	2.8	1.3
Other	4.4	5.3
No treatment	1.3	8.1
Only hydrocortisone	–	1.6

^a^Up to five answers were possible.

### Comparison of time to diagnosis and persistence of symptoms

To compare the time to diagnosis and the persistence of symptoms following treatment, an analysis of a number of variables was performed comparing the group with persistent symptoms after treatment (*n*  = 283) with those who did not (*n*  = 37) in terms of time to diagnosis. Patients with a longer time to diagnosis reported significantly more frequent weight gain (*P* = 0.008), and more frequent reduced libido (*P* = 0.03) after treatment. Although not statistically significant, there was a strong trend towards patients reporting a longer time to diagnosis and a greater frequency of persistent perceived bone issues after treatment (*P* = 0.053), as well anxiety (*P* = 0.07) and depression/mood concerns (*P* = 0.08).

### Physicians involved in follow-up

Once diagnosed, almost all patients (97.8%, *n* = 313) were managed by an endocrinologist, followed by a GP/family doctor (56.3%, *n* = 180). A psychiatrist/psychologist was involved in 23.4% (*n*  = 75), followed by a physiotherapist (14.4%, *n* = 46), rheumatologist (14.4%, *n* = 46), gynecologist (14.1%, *n* = 45), cardiologist (13.4%, *n* = 43), dermatologist (11.6%, *n* = 37) and a dietician (9.7%, *n* = 31) (Table 3).

### Treatment of persistent symptoms

Table 4 shows the prevalence of persistent symptoms after treatment, common ongoing comorbidities included fatigue, muscle weakness and weight gain. The percentage of patients who were treated for comorbidities is also shown. Noticeable undertreatment occurred for many symptoms, for example, fatigue was a consistent symptom for 66.3% (*n*  = 212), whereas only 15.9% (*n*  = 51) were receiving ongoing care for fatigue and persistent muscle weakness was reported in 48.8% (*n*  = 156) with 17.2% (*n*  = 55) of patients being treated for this (Table 4).

The high frequency of persistent symptoms suggests that patients were not followed-up by specific specialists, for example of the 212 patients with persistent fatigue, only 60 (28.2%) were seeing a psychiatrist/psychologist (Table 4). Enduring poor concentration and memory problems were relatively frequent (35.9%, 33.8%) but were rarely treated by a specialist (4.1 and 5.6%, respectively).

Three-quarters of patients reported that their work life had been affected (75%, *n* = 240). Social life (65.3%, *n* = 209), family life (57.8%, *n* = 185), interpersonal relationships (51.6%, *n* = 165), and sexual life (48.8%, *n* = 155) had also been significantly affected by their illness. Thirty-seven percent of the patients (*n*  = 118) reported that their economic situation had been negatively affected. ‘Other’ responses for this question included reductions in self-esteem, self-image and self-confidence. Sixty-three percent of patients (193/305) were satisfied with their treatment and 36.7% (*n*  = 112) were not.

### Comparative analysis physician questionnaire

In the complementary physician questionnaire (*n*  = 40), unlike the patient questionnaire where most respondents were from the United Kingdom, the United States of America, the Netherlands and France, most of the physicians surveyed were from Western Europe, although there were representatives from other parts of the world. In the physician questionnaire, 83% (*n*  = 33) were endocrinologists, 13% (*n*  = 5) internal medicine specialists and 5% (*n*  = 2) other disciplines. Sixty percent (*n*  = 24) had over 10 years clinical experience, and 93% (*n*  = 37) were experienced in the treatment of CS, seeing an average of 10 patients per year. Of the specialities involved in the care of CS, 96% of physicians (*n*  = 38) considered endocrinologists to be involved, 48% (*n*  = 19) included family doctors/GPs, 20% (*n*  = 8) cardiologists, 28% (*n*  = 11) psychiatrists/psychologists and 28% (*n*  = 11) included dieticians. These results are consistent with the patients’ perceptions, with the exception of dieticians, who only 10% of patients reported seeing (Table 3).

**Figure 2:**
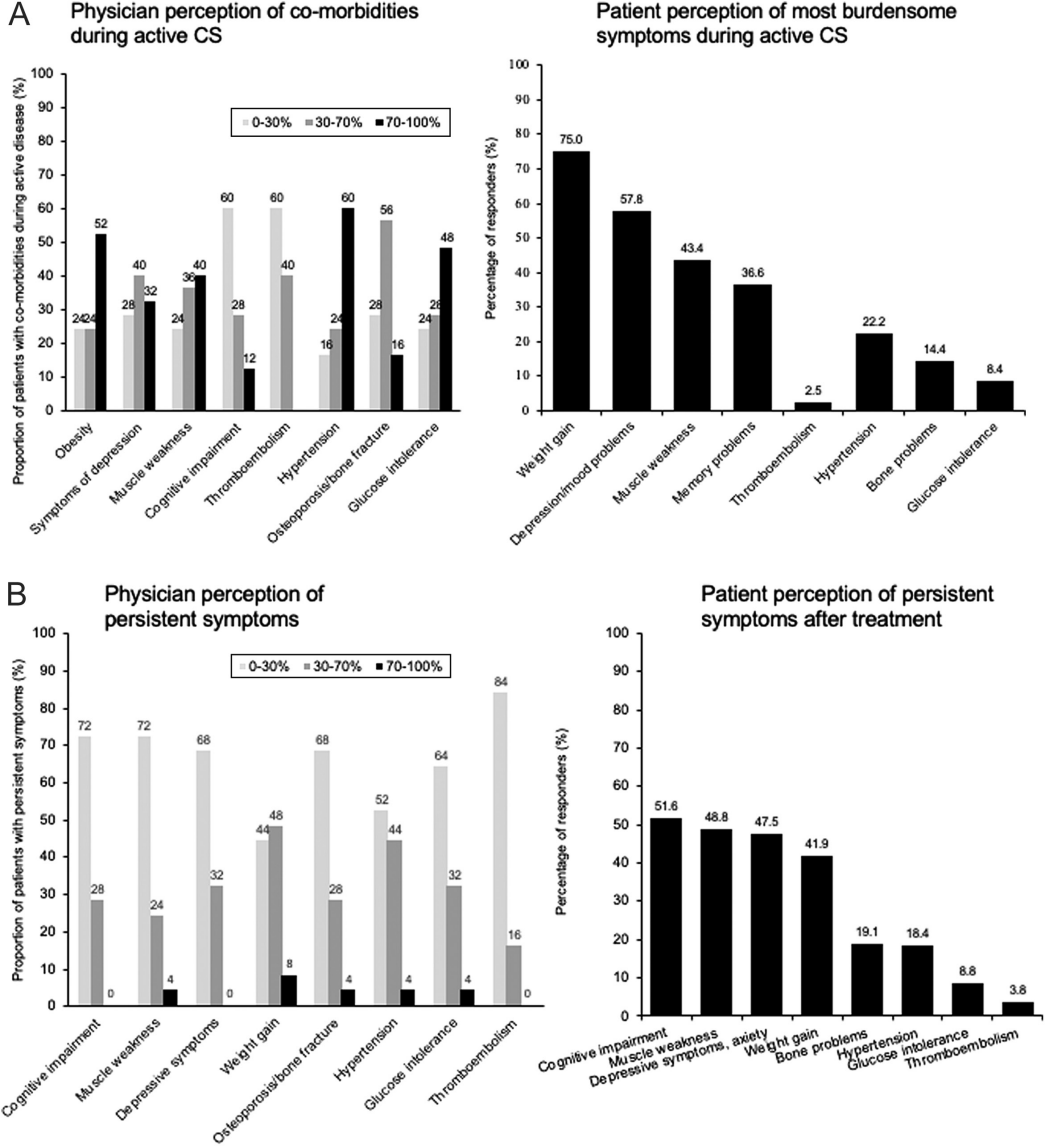
(A) Physician (*n* = 40) perception of patient comorbidities (left) and patient reports of the most burdensome symptoms during active CS (right). (B) Physician (*n* = 40) perception of CS symptoms after cure (right) and patient reports of persistent burdensome symptoms after treatment (left). Only the relevant common results from the physician and patient surveys are shown above. The physician survey included categories ‘insulin resistance’, ‘dyslipidaemia’, ‘cardiovascular complications’ and ‘psychosis’, which are not shown because these same categories were not reported in the patient survey. In the patient survey, responses for the categories: ‘anxiety’ were regrouped with ‘depressive symptoms’ and ‘memory problems’ and ‘poor concentration’ were regrouped into the ‘cognitive impairment’ category for easier comparison with the physician survey.

Figure 2A compares the frequency of common symptoms that patients found to be most burdensome during the active phase of the disease, with what physicians thought were the most common symptoms. Although for methodological reasons a statistical comparison was not possible and the comparisons are approximate, these findings suggest that physicians’ perceptions of the prevalence of symptoms were different from those reported by patients. A majority of physicians (Fig. 2A) inadequately estimated (both underestimated and overestimated) the presence of depression, muscle weakness, cognitive impairment, hypertension, bone problems and glucose intolerance. Figure 2B compares the physician’s perception of the frequency of persistent symptoms with the patients’ experience of persistent symptoms. A majority of physicians differently estimated the prevalence of persistent cognitive impairment, muscle weakness, depressive symptoms and weight gain.

## Discussion

This large, international CS patient survey confirms previous findings that despite complaining of multiple symptoms, there is a mean 34-month delay in diagnosis (9). In addition, despite treatment resulting in biochemical remission, patients report persistent comorbidities with associated psychological and social impacts that negatively affect the QoL (11, 12). In the present survey a majority of patients reported that they are not being managed by the appropriate specialists, suggesting an absence in multidisciplinary care that may be secondary to an underestimation of the sequelae of CS by endocrinologists.

The present survey confirmed that no specific symptom initiated a diagnosis, but rather a range of burdensome symptoms occurring with similar frequency to those reported in previous surveys (1, 2), with the notable difference in that in a USA-German survey, cognitive and psychological symptoms were bothersome for 61% of US and 66% of German patients (13), whereas in the present survey 38% considered depression/mood problems burdensome. Such differences may be a result of different terms being used to describe depression or mood symptoms as well as cultural differences between populations.

The distribution of time to diagnosis, with around 50% diagnosed after 2 years of symptoms and approximately 30% still undiagnosed after 3 years is of a similar magnitude to previous surveys, where 67% of patients waited at least 3 years until diagnosis (14). In the CSFR study in 2014, patients waited a median of 5 years until diagnosis (15). Even though the estimated time to diagnosis may be similar to those in previous studies – 34 months a recent meta-analysis (9) and 2 years in the ERCUSYN database (16) – there is clearly still room for improvement, especially as delayed diagnosis is associated with persistent comorbidities (9, 17, 18, 19). Physicians should consider that in patients with diabetes, hypertension and osteoporosis hypercortisolism may be hidden (20). Due to the elevated incidence of mood and cognitive dysfunction at CS diagnosis, questioning the patient whether they feel that ‘something unusual is happening’ such as mood swings and sleeping disorders may be helpful, as a not insignificant proportion of patients self-diagnose CS (15).

Awareness of the clinical presentation patterns of CS should be increased among general practitioners but also in specialists other than endocrinologists. In the current survey, the low proportions of physiotherapists, neurologists, orthopaedic surgeons and psychiatrists identifying CS represent an educational opportunity to improve early diagnosis. It is for instance not widely known that venous thromboembolic events or fragility fractures can be a presenting symptom of CS (20, 21). It is encouraging that rheumatologists already recommend excluding occult endogenous hypercortisolism as a first cause of muscle weakness (22).

Multidisciplinary care is recommended for the ongoing management of patients after biochemical cure, with a particular emphasis on the QoL, depressive symptoms and anxiety (11). Specialist care is recommended for specific comorbidities, for example physiotherapists are required to help revert musculoskeletal impairment and prevent further deterioration (23), and bone specialists are required to manage the individual patient fracture risk according to the patient’s age and evolution of bone status after surgery (24). In the present survey, almost all patients were treated by endocrinologists and the role of specialists treating particular comorbidities was limited despite the ongoing complaints in patients. This is particularly evident in the high prevalence of muscle weakness, which was rarely managed by physiotherapists. This failure to provide multidisciplinary care may account for why nearly 40% of CS patients were dissatisfied with their treatment.

The exact number of patients with controlled hypercortisolism cannot be evaluated from the questionnaire. The degree of control of hypercortisolism remains debatable in patients treated with cortisol-lowering agents and may not be equivalent to remission following surgery (25, 26). In the present survey, the vast majority reported persistent and burdensome symptoms despite treatment, which is in line with previous reports of persistent low body satisfaction and high rates of depression and anxiety (27). When compared with longer time to diagnosis, the only comparisons that reached statistical significance were weight gain and decreased libido; whereas, there was a trend towards extended time to diagnosis and worsening of depressive symptoms and anxiety. These findings confirm the need for early diagnosis and treatment as the duration of exposure to hypercortisolism is a predictor of persistent morbidities and long-term impairments in the QoL (15).

Although the parallel physician perception questionnaire was limited by small size and methodological differences in comparison to the patient survey, the results suggest that physicians’ perceptions contrast with patients’ experiences. Physicians tended to underestimate weight gain and cognitive impairment during the active phase of the disease, and underestimate the prevalence of cognitive impairment, depressive symptoms and muscle weakness following treatment. A recent survey on physician vs patient perspectives on postsurgical recovery also highlighted important differences in perceptions, suggestive of poor communication (28). However, these comparisons are limited in that physicians’ estimations may be influenced by the clinical importance of certain symptoms, whereas for patients these may or may not be particularly onerous. Nevertheless, these findings do suggest that some symptoms do not receive enough attention, possibly due to insufficient awareness of these symptoms as real clinical problems.

The strength of this survey is that it includes a large and international population, whereas previous surveys tended to be carried out in individual countries. It informs the quantitative and qualitative understanding of CS patients’ experiences with their treatment journeys and highlights some important lacunae in the management of CS, as well as identifying some differences in physician and patient perceptions about the burden of CS comorbidities.

A limitation in the study design was the inability of the questionnaire to clearly distinguish a subgroup who were biochemically cured and had ongoing symptoms. Indeed, remission was based on patients’ declarations instead of an objective hormone assessment, which is an unavoidable limitation of online surveys. On the other hand, the survey was precisely designed to capture patients’ perceptions about their health status, regardless of having received a diagnosis of “remission” or not from their endocrinologist. Patients who had pituitary surgery were not considered as being “in remission” in order to mitigate the impact of this limitation on the final analysis. The major limitations of this survey also include its cross-sectional design, depending upon an individual assessment at a single time point and relying on patients’ memories. The comparison of the patient and doctor cohorts was limited by having different questionnaire methodologies and the lack of matching of patients and their endocrinologists. The questionnaire results could also not be corroborated against clinical records and no matched control group was assessed. Selection basis was another potential limitation, as patients were recruited through patient associations, which may have skewed the population towards patients with a higher disease burden; moreover, patients with chronic conditions who respond to questionnaires tend to have a low QoL (15).

## Conclusion

This international cross-sectional study confirms that symptoms experienced by patients with CS are diverse, burdensome and endure beyond treatment (20). Delays in diagnosis may contribute to persistent symptoms after treatment. Care of patients with persistent comorbidities affecting the QoL (e.g. obesity, cognitive impairment, depression and muscle weakness) could be improved through more frequent multidisciplinary collaboration with healthcare professionals outside of endocrinology.

## Supplementary Material

Supplementary Material

## Declaration of interest

A T participated in research studies, received research grants and honorarium for talks at symposia and boards from HRA Pharma Rare Diseases, Pfizer, Novartis and Recordati Rare Diseases. C A participated in research studies and received honoraria for talks at symposia and participation in advisory boards from HRA Pharma Rare Diseases. E V participated in research studies and received honoraria for talks at symposia and participation in advisory boards from HRA Pharma Rare Diseases and Recordati Rare Diseases. I C is an investigator in studies using relacorilant (Corcept Therapeutics) in patients with hypercortisolism and has received consulting fees from Corcept Therapeutics and HRA Pharma Rare Diseases. R F has received research grants from Strongbridge and Recordati Rare Diseases and honoraria for talks at symposia and for participating in advisory boards from HRA Pharma Rare Diseases, Corcept, Ipsen, Novartis and Recordati Rare Diseases. M A H and S I are employees of HRA Pharma Rare Diseases. R A F is a member of the editorial board of Endocrine Connections. He was not involved in the editorial or review process of this paper, on which he is listed as an authors.

## Funding

This work did not receive any specific grant from any funding agency in the public, commercial or not-for-profit sector.
