# Supplementary material for: Unmet needs in Cushing’s syndrome: the patients’ perspective
Source: Endocr Connect. 2022 May 27;11(7):e220027. doi: 10.1530/EC-22-0027 (PMC9254293; doi:10.1530/EC-22-0027)
Supplement: Supplementary Material [file supplementary_material.pdf]

11. Appendix

Cushing's syndrome: Patient questionnaire

This survey is intended for patients only.

We are interested in understanding more about Cushing's syndrome from patients like you, including your experience with diagnosis and treatment, and the impact the disease has had on your life. We would be grateful if you could complete this survey, and your answers will be reviewed by an expert faculty and possibly published in a medical journal to help those treating the disease understand and improve the quality of care for people living with Cushing's syndrome. In order for your voice to be heard, every answer counts.

Your responses will be confidential, and we will not collect identifying information such as your name, email address or IP address. All data are stored in a password-protected electronic format.

Country: [Drop-down menu]

Age:

i. Under 18 years old
ii. 18–24 years old
iii. 25–34 years old
iv. 35–44 years old

v. 45–54 years old

vi. 55–64 years old

vii. 65–74 years old

viii. 75 years or older

ix. Prefer not to say

Sex:

i. Male

ii. Female

iii. Prefer not to say

Highest educational degree:

i. High school graduate or diploma

ii. Undergraduate degree

iii. Post-graduate degree

iv. Prefer not to say

Before a diagnosis of Cushing's syndrome was made:

1. What signs/symptoms did you first notice? Please select all that apply:

    i. Fatigue
    ii. Obesity/weight gain
    iii. Skin problems (thin skin, easy bruising)
    iv. Muscle weakness
    v. Sugar (glucose) intolerance
    vi. High blood pressure (hypertension)
    vii. Bone problems and fragility
    viii. Unwanted hair growth (hirsutism)
    ix. A blood clot (venous thrombosis, pulmonary embolism)
    x. Decreased libido/sex drive
    xi. Depression, mood problems
    xii. Anxiety
    xiii. Lack of attention/concentration
    xiv. Memory problems
    xv. Sleep disturbances
    xvi. Infections
    xvii. Disturbances with your periods/menstrual cycle
    xviii. Acne
    xix. Others (please specify………)

2. What signs/symptoms were most burdensome for you? Please select all that apply and specify the reason for each symptom chosen:

i. Fatigue (why? ………)

ii. Obesity/weight gain (why? ………)

iii. Skin problems (thin skin, easy bruising) (why? ………)

iv. Muscle weakness (why? ………)

v. Sugar (glucose) intolerance (why? ………)

vi. High blood pressure (hypertension) (why? ………)

vii. Bone problems and fragility (why? ………)

viii. Unwanted hair growth (hirsutism) (why? ………)

ix. A blood clot (why? ………)

x. Decreased libido/sex drive (why? ………)

xi. Depression, mood problems (why? ………)

xii. Anxiety (why? ………)

xiii. Lack of attention/concentration (why? ………)

xiv. Memory problems (why? ………)

xv. Sleep disturbances (why? ………)

xvi. Infections (why? ………)

xvii. Disturbance with your periods/menstrual cycle (why? ………)

xviii. Acne (why? ………)

xix. Others (please specify………)

3. How long did it take between reporting the first signs/symptoms to your doctor and a diagnosis being made? Please select one option:

i. 0–6 months

ii. 6–12 months

iii. 1–2 years

iv. 2–3 years

v. Over 3 years (please specify………)

4. Which physician first suspected Cushing’s syndrome and/or prescribed the first tests that confirmed the disease? Please select one option:

i. Endocrinologist (specialist in hormonal diseases)

ii. Primary care/family doctor

iii. Cardiologist (heart specialist)

iv. Oncologist (cancer specialist)

v. Haematologist (specialist in blood disorders)

vi. Gynaecologist (specialist in the female reproductive system)

vii. Dermatologist (skin specialist)

viii. Bone specialist

ix. Ophthalmologist (eye specialist)

x. Psychiatrist (mental health specialist)

xi. Other (please specify………)

Once the diagnosis of Cushing’s syndrome was made:

5. Since diagnosis, what type of physicians/healthcare professionals have been involved in helping to manage your disease? Please select all that apply:

i. Endocrinologist (specialist in hormonal diseases)

ii. Primary care/family doctor

iii. Cardiologist (heart specialist)

iv. Oncologist (cancer specialist)

v. Haematologist (specialist in blood disorders)

vi. Gynaecologist (specialist in the female reproductive system)

vii. Dermatologist (skin specialist)

viii. Bone specialist

ix. Psychiatrist/psychologist (mental health specialists)

x. Social worker

xi. Physiotherapist

xii. Dietitian

xiii. Other (please specify………)

6. What is your current situation with regards to treatment?

Please select one option:

i. In remission (no treatment)

ii. On treatment with cortisol-lowering medication

iii. Received an operation to remove your adrenal glands

iv. Received an operation on your pituitary gland

v. Received radiotherapy to treat your pituitary gland

vi. Other (please specify………)

7. Following your treatment for Cushing's syndrome, do you still experience any symptoms related to the condition?

i. No

ii. Yes (please choose up to five symptoms that are the most burdensome for you)

i. Fatigue

ii. Obesity/weight gain
iii. Skin problems (thin skin, easy bruising)
iv. Muscle weakness
v. Sugar (glucose) intolerance
vi. High blood pressure (hypertension)
vii. Bone problems and fragility
viii. Unwanted hair growth (hirsutism)
ix. Problems with a blood clot
x. Decreased libido/sex drive
xi. Depression, mood problems
xii. Anxiety
xiii. Lack of attention/concentration
xiv. Memory problems
xv. Sleep disturbances
xvi. Infections
xvii. Disturbances with your periods/menstrual cycle
xviii. Acne
xix. Others (please specify………)

8. Do you still receive treatment for any of the following symptoms? Please select all that apply, and if yes, please specify for each symptom:

i. Fatigue (please specify………)
ii. Obesity/weight gain (please specify………)
iii. Skin problems (thin skin, easy bruising) (please specify………)
iv. Muscle weakness (please specify………)

v. Sugar (glucose) intolerance (please specify………)

vi. High blood pressure (hypertension) (please specify………)

vii. Bone problems and fragility (please specify………)

viii. Unwanted hair growth (hirsutism) (please specify………)

ix. Problems with a blood clot (please specify………)

x. Decreased libido/sex drive (please specify………)

xi. Depression mood problems (please specify………)

xii. Anxiety (please specify………)

xiii. Lack of attention/concentration (please specify………)

xiv. Memory problems (please specify………)

xv. Sleep disturbances (please specify………)

xvi. Infections (please specify ………)

xvii. Disturbances with your periods/menstrual cycle (please specify………)

xviii. Acne (please specify………)

xix. Others (please specify………)

9. Did you receive medication to prevent or treat problems with a blood clot (venous thrombosis, pulmonary embolism) before and after your operation? If so, how long were you treated for? Please select one option:

i. Yes (please specify duration of treatment………)

ii. No

iii. I don’t know

10. Which areas of your life have been most affected by your illness? Please select all that apply:

iv. Work
v. Economic situation
vi. Family life
vii. Relationships
viii. Sex life
ix. Social life
x. Other (please specify………)

11. Do you have any questions about your disease that you feel have not been fully answered?

………

12. Having now been diagnosed and treated, are you satisfied with your treatment? Please select one option:

i. Yes (please provide further details as to why it has been satisfactory)

………

ii. No (please provide further details as to why it has not been satisfactory)

………
